# What is known about adolescent dysmenorrhoea in (and for) community health settings?

**DOI:** 10.3389/frph.2024.1394978

**Published:** 2024-07-23

**Authors:** Sharon Dixon, Jennifer Hirst, Neda Taghinejadi, Claire Duddy, Katy Vincent, Sue Ziebland

**Affiliations:** ^1^ Nuffield Department of Primary Care Health Sciences, University of Oxford, Oxford, United Kingdom; ^2^ Nuffield Department of Women’s and Reproductive Health, University of Oxford, Oxford, United Kingdom

**Keywords:** adolescent, dysmenorrhoea, period pain, primary care, general practice, narrative synthesis, uncertainties

## Abstract

**Introduction:**

Dysmenorrhoea affects many adolescents with significant impacts on education and well-being. In the UK, most of the adolescents who seek care (and many never do), will do so through general practice (primary care). Knowing how best to care for adolescents reporting menstrual pain is an area where UK general practitioners would like better guidance and resources.

**Methods:**

This mixed-methods narrative synthesis collates community and specialist evidence from 320 papers about adolescent dysmenorrhoea, with a UK general practice community health perspective.

**Results:**

We report a narrative summary of symptoms, cause, consequences and treatments for adolescent dysmenorrhoea. We highlight areas of tension or conflicted evidence relevant to primary care alongside areas of uncertainty and research gaps identified through this synthesis with input from lived experience advisers

**Discussion:**

There is little evidence about primary care management of adolescent dysmenorrhoea or specific resources to support shared-decision making in general practice, although there are evidence-based treatments to offer. Primary care encounters also represent potential opportunities to consider whether the possibility of underlying or associated health conditions contributing to symptoms of dysmenorrhoea, but there is little epidemiological evidence about prevalence from within community health settings to inform this. The areas where there is little or uncertain evidence along the care journey for adolescent dysmenorrhoea, including at the interface between experience and expression of symptoms and potential underlying contributory causes warrant further exploration.

**Systematic Review Registration:**

https://www.crd.york.ac.uk/PROSPEROFILES/256458_STRATEGY_20210608.pdf, identifier (CRD42021256458).

## Introduction

Dysmenorrhoea (menstrual pain) affects many adolescents who menstruate around the world, with potential adverse impacts on health, well-being, school, leisure, and work engagement (1). While dysmenorrhoea can occur in the absence of demonstrable underlying conditions, it can also be part of the symptom experience associated with conditions such as endometriosis, ovarian cysts, congenital genital tract developmental anomalies or pelvic inflammatory disease (2).

Our qualitative study with general practitioners working in England exploring how they navigate supporting patients with possible endometriosis elucidated their concern and uncertainty about how best to care for adolescents with dysmenorrhoea. This highlighted concerns about the lack of evidence about long-term outcomes and whether interventions in adolescence influence outcomes in adulthood. They identified a need for primary-care focussed evidence (3), a need this synthesis seeks to address.

This synthesis aims to document relevant evidence about adolescent dysmenorrhoea settings to create an evidence-based summary with a general practice focus. While this review does not focus on adolescent endometriosis, it considers the interface between associated health conditions and adolescent dysmenorrhoea in the context of presentation to primary care. We also sought to identify potential, or suggested, research gaps.

The majority of health contacts in the UK occur in general practice (4), typically the first port of call when people are presenting with symptoms and seeking care or treatment (5). In addition, general practice holds a gatekeeper function, coordinating referrals for more specialist investigations and care (6). This means that the population incidence of many conditions will be different in community and specialist care settings, risking a denominator error when data are extrapolated between them. This synthesis seeks to recognise this uncertainty and collate evidence from both community and specialist settings with a community health perspective.

PROSPERO 2021 CRD42021256458.

## Method

We undertook an integrative mixed methods synthesis (7, 8), reported as a narrative summary (9). The search strategy, approach taken to data selection, data extraction, and analysis is described in full in the study protocol. https://www.crd.york.ac.uk/PROSPEROFILES/256458_STRATEGY_20210608.pdf.

### Search strategy

We conducted an exhaustive bibliographic search for: dysmenorrhoea, period pain, teenager, and adolescent. We conducted a parallel targeted purposive search using the terms adolescent, teenagers, dysmenorrhoea, and endometriosis. We searched the following databases: MEDLINE, Embase, PsycINFO, CINAHL and ERIC. We included all papers written in the English language. Our search strategy is available at: https://www.crd.york.ac.uk/PROSPEROFILES/256458_STRATEGY_20210608.pdf.

### Selection and appraisal of documents

All study designs and typologies, including editorials and opinion pieces were considered for inclusion if they offered information that could inform the study research aim of documenting and assimilating existing evidence underpinning diagnosis and care of adolescents experiencing dysmenorrhoea in community settings. Because of the lack of direct primary care evidence, we adopted a broad inclusion strategy. We did not apply any date range or regional setting limits on data inclusion. We only included papers where the mean age of participants was between ten and nineteen, in line with the World Health Organisation definition of adolescence (10).

We included papers written in English and relevant to the context of UK general practice. Because complementary and alternative medications (treatments not offered in mainstream health settings in the UK, but which may be accessed alongside or instead of these services, such as homeopathy and chiropractic treatments) are not routinely accessible to or provided by UK NHS general practice (11), we did not include primary evidence documenting these. Whilst acupuncture is not routinely available in NHS or primary care clinics, it may be available or recommended within specialist settings, and so we included systematic reviews detailing evidence about this in adolescence. We included systematic reviews about treatments sanctioned in UK guidance if they included adolescent participants. We did not include studies of medications unavailable in the UK (listed in the British National Formulary).

The review team (SD, CD, NT) developed, piloted, and agreed a data extraction tool. All abstracts identified by searches were uploaded to Rayyan systematic review software. SD reviewed all abstracts, with twenty percent co-checked by CD/NT. We had a protocol plan for escalation for any disagreements or differing opinions or perspectives, but did not need to utilise this strategy. We had a shared understanding of the aim which was to document evidence and actively consider research gaps, and so we included any studies reporting menstrual pain during adolescence, and this broad inclusion facilitated our wide inclusion and minimised disagreements.

### Data extraction and analysis

All full text documents were appraised by SD, with twenty percent checked by CD/NT. The wide approach we took to inclusion resulted in minimal discrepancies. All full text documents appraised were coded within NVivo 12. To encompass and represent the breadth of evidence included, SD undertook a thematic analysis of the narrative of the written studies and papers. Our final coding framework is represented as the thematic headings described in Table 1. Having identified the disparate nature of the studies, including variance of study type, geographical setting, chronological period, and nature of enquiry, along with the preponderance of surveys where the survey tool was not available, we elected to not formally undertake quality appraisal, but to instead adopt a broad inclusion approach for all potentially relevant studies and assimilate them into the descriptive analysis. We have documented and mapped the focus, typology and setting of each included paper (Table 2).

**Table 1:** Describes the thematic categories within which we present and report our findings.

Theme	Sub-themes
What is dysmenorrhoea?	Core symptoms
	Associated symptoms
	pathogenesis and classification of dysmenorrhoea
How common is dysmenorrhoea?	Prevalence and incidence
What might contribute to dysmenorrhoea (epidemiological associations)	Menstrual associations
	Non-menstrual associations
	What conditions may be associated with dysmenorrhoea?
What impacts does dysmenorrhoea have on adolescents?	Education and work
	Sport and leisure activities
	Well-being and quality of life
	Long-term outcomes
What do we know about how and when adolescents access care for dysmenorrhoea?	Accessing information
	Accessing health services
When might dysmenorrhoea signal the presence of a contributory cause?	Congenital developmental malformations
	Endometriosis
	Other causes of secondary dysmenorrhoea
What treatments might help?	Self-care
	Non-pharmacological
	Pharmacological
Uncertainties	Tensions and inconsistencies
	Research or knowledge gaps

**Table 2:** Summarises the focus of studies included in this review.

Focus of studies included in final synthesis:	
**Dysmenorrhoea study characteristics**	**Number of papers**
Background review/expert opinion or guidance	22
Epidemiology or associated conditions	163
Evidence about treatment	58
Qualitative studies	9
**Secondary dysmenorrhoea**	**Number of papers**
Endometriosis and dysmenorrhoea	33
Congenital and developmental anomalies and dysmenorrhoea	35
**Study setting (participants recruited from)**	**Number of papers**
Community/school	153
Primary care/community health	9 (3 primary care, 3 teen health clinic, 3 community sexual health & contraception)
Specialist care	94
**Study type**	
Expert opinion/review	33
Systematic review	24
Observational study (cohort, cross-sectional survey, observational anthropometrics)	152
Non RCT interventional study	19
RCT	14
Case study/case report	64
Qualitative study	13

### Synthesis reporting

We report this narrative synthesis in thematic categories using illustrative references. These thematic categories are summarised in Table 2. These may not represent all of the references that contributed to the theme. The full reference list of studies that contributed evidence to each theme within this synthesis is included in Supplementary Appendix A.

Our included population is adolescents who menstruate. The majority of the studies we identified used gendered terms such as girls or women in describing their work, including in relation to study populations and when reporting findings. We identified one paper reporting specifically on endometriosis and dysmenorrhoea amongst trans-male adolescents. We recognise that gendered terms do not represent all experiences of menstruation.

### Patient and public involvement

This project is under-pinned by advice from a PPI group including adolescents with dysmenorrhoea and adults with lived experience of endometriosis and of adolescent dysmenorrhoea. They have assisted with interpreting findings, and in identifying potential gaps and research needs.

## Results

**Figure 1:**
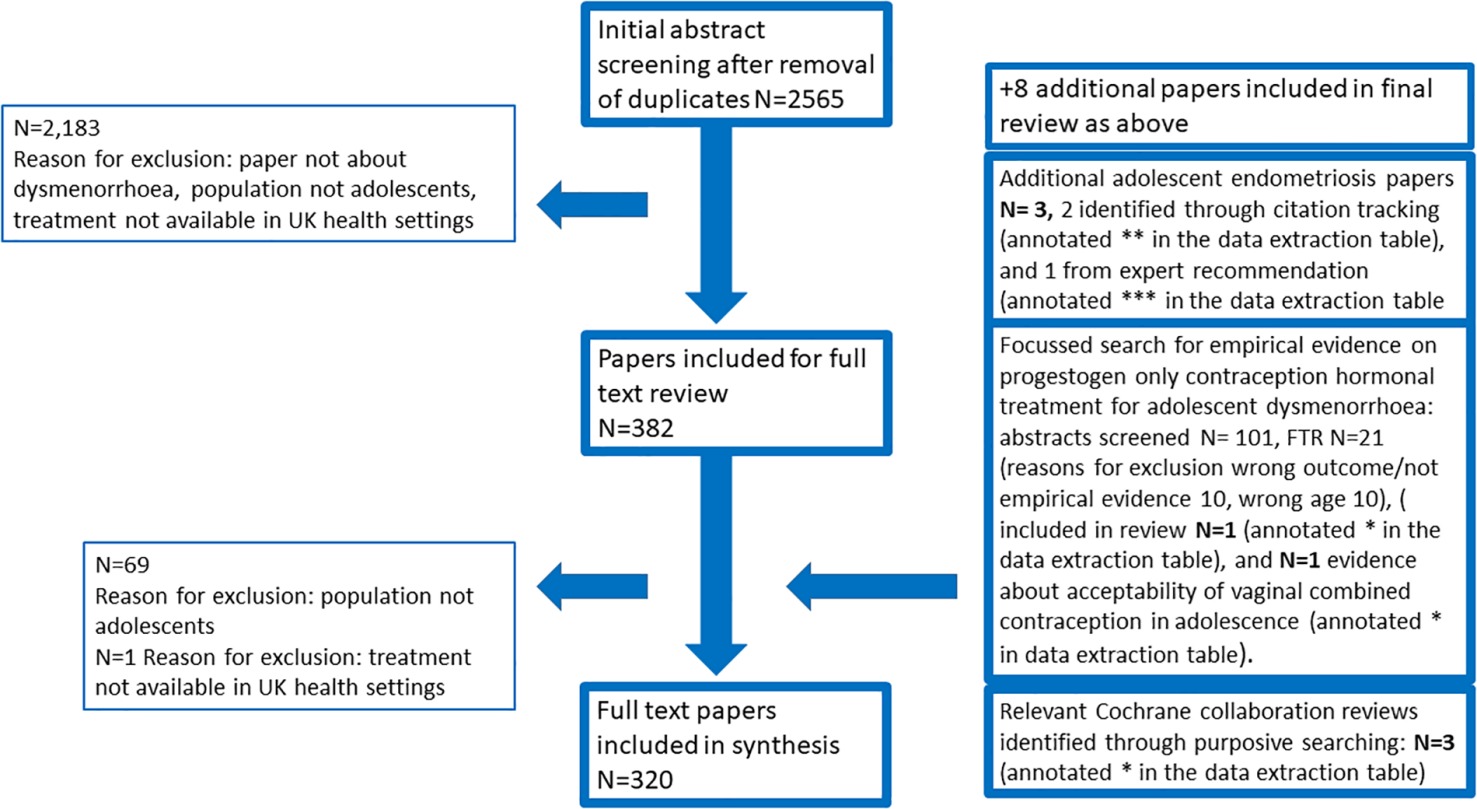
Summarises the PRISMA flowchart for the review.

Our search yielded 2,565 unique abstracts. Following abstract screening, 382 papers were included for full text review, and 312 were included in the final synthesis. In addition, in line with our protocol, we added 6 papers from citation tracking (*N* = 2), purposive sampling (*N* = 3) and expert recommendation (*N* = 1). Noting an evidence gap, we undertook a detailed search to look for evidence about progestogen-only contraception and adolescent dysmenorrhoea, and included 2 studies from this search. These are annotated within the data extraction table, using the legend described in the PRISMA diagramme. Figure 1 summarises the search strategy and reasons for exclusion. Table 1 summarises and characterises the included papers. Supplementary Appendix A includes the data extraction sheet detailing all included studies. The study dates ranged from 1957 to 2023. Table 1 demonstrates the balance of evidence generated between community and specialist health settings and the relative lack of evidence from within community health settings. This demonstrates the relative lack of randomised controlled trial evidence and qualitative research within adolescents, although they are a population with both high prevalence and symptom burden of dysmenorrhoea. Table 2 details the thematic categories in which we present this narrative synthesis findings.

### What is dysmenorrhoea?

#### Core symptoms

Dysmenorrhoea is a descriptive symptomatic term for the pain associated with menstruation. The term derives from the Greek words of dys (difficulty, pain or trouble), menos (month) and rrhoea (flow) (12, 13). The pain may be experienced as cramping, sharp, stabbing, aching, shooting or constant pains through the lower abdomen, pelvic, and inguinal region. Pain may also be experienced in the back and thighs. Pain typically begins between 2 and 3 days before and after the onset of bleeding, most commonly on the first day of bleeding (14–17).

#### Associated symptoms

Adolescents often experience extra-pelvic symptoms as part of dysmenorrhoea. These include tiredness, gastrointestinal symptoms [nausea, vomiting, changes in bowel habit (diarrhoea or constipation), altered appetite], bloating (oedema), dizziness, headaches, breast pain, insomnia and emotional changes and lability (mood changes, low mood, reduced concentration, irritability). For most affected adolescents, symptoms start with menstruation and last 1–3 days from onset (14–37). Some papers using participant experience to delineate symptoms associated with dysmenorrhoea also report pelvic pain at non-menstrual times of the cycle, suggesting that dysmenorrhoea can be associated with acyclic pain (mid-cycle and acyclic pain) (1, 21, 34, 38–40).

#### Pathogenesis and classification of dysmenorrhoea

While previously regarded as a maladaptive response to menstruation (41–44), and as a psychosomatic or psychosocial phenomenon (45–50), evolving understanding contributed to a re-framing of dysmenorrhoea as a physiological (medical) entity (42, 51–59). Developments in understanding the pathogenesis of menstrual pain elucidated the role of prostaglandins and leukotrienes in menstrual pain (56, 57, 60, 61), although it is sometimes described as a “learned behaviour” (62). Studies do not show a consistent association between uterine morphology and dysmenorrhoea (63), although utero-cervical angle may predict severity (64).

As understanding of dysmenorrhoea has evolved, the ways in which it is categorised have also changed. Dysmenorrhoea is now typically categorised into primary dysmenorrhoea (no known or discernible underlying cause), and secondary dysmenorrhoea (menstrual pain in association with an identified underlying condition such as pelvic anatomical pathology). Causes of secondary dysmenorrhoea in adolescence include endometriosis, congenital developmental anomalies of the genitourinary tract, ovarian cysts, and pelvic inflammatory disease (2, 65, 66). Historically, primary dysmenorrhoea was further categorised as “spasmodic” (sometimes called true) dysmenorrhoea (cramping pains within first 48 h of onset of menstruation) (67) or “congestive” (more widespread bodily aches) (68). While this has largely now been abandoned (13), the terms do still sometimes appear (and sometimes interchangeably) (69) in the literature and so are mentioned here.

### How common is dysmenorrhoea?

#### Prevalence and incidence

**Figure 2:**
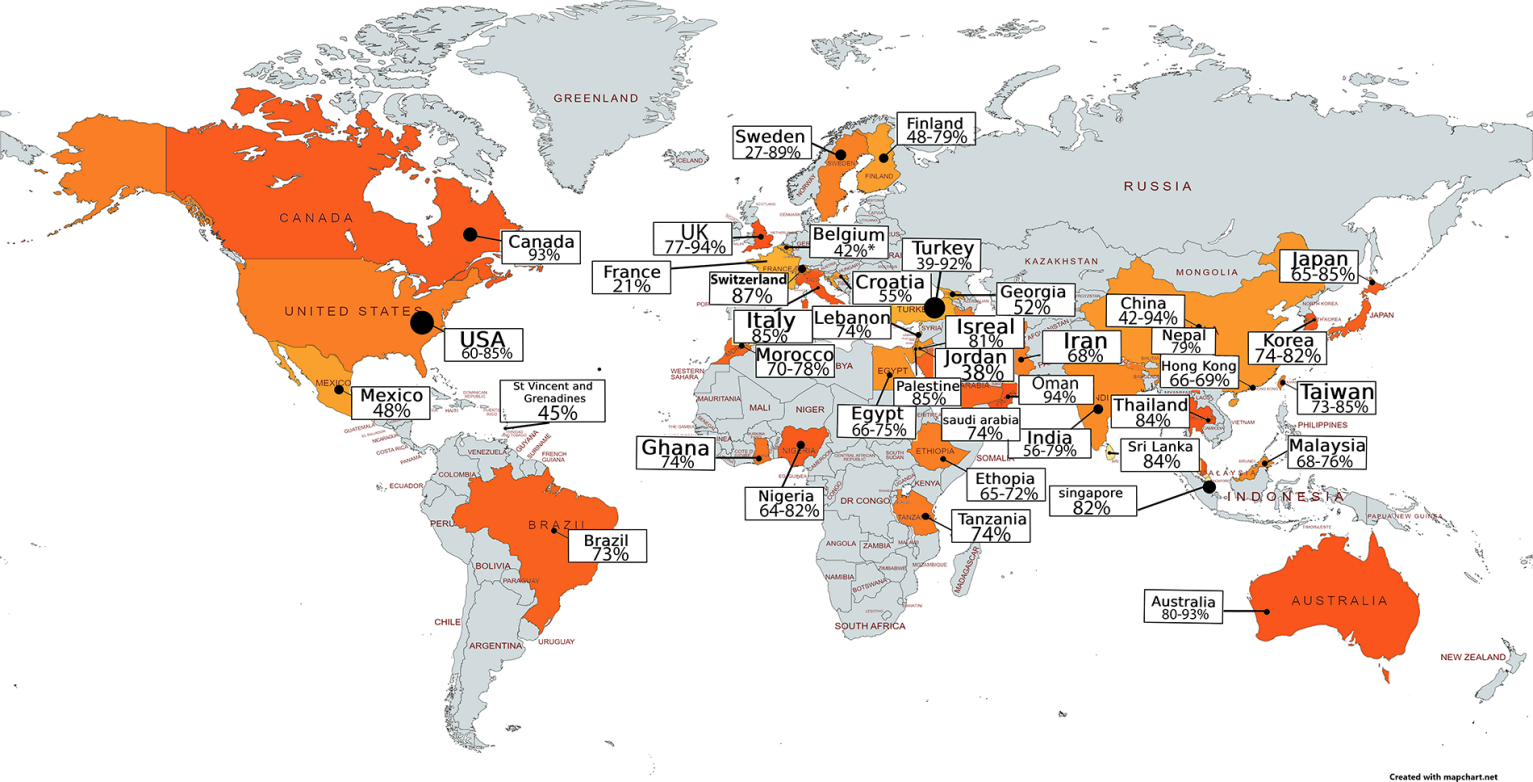
Recognising the range of settings, temporal context, measuring scales and approaches, and aims of the included studies, we have not quantitatively assimilated the findings about dysmenorrhoea prevalence in community settings, but have visually mapped the figures for prevalence in the evidence we identified.

Data on the prevalence of dysmenorrhoea around the world is hard to compare directly, because it has been collected in a variety of settings, using different approaches, and with young people of different ages, and from different cultural contexts. There are studies that demonstrate different rates of dysmenorrhoea between rural and urban contiguous settings (70–74) or between different ethnic groups in one geographical setting (74–76). Menstrual stigma (secrecy) may also influence reporting of dysmenorrhoea (77–80), although this may be evolving with increasing recognition of menstrual health needs, making the year when the study was undertaken a relevant contextual factor. In the studies we identified that reported dysmenorrhoea prevalence, we observe a trend towards higher prevalence over time. Recognising the range of settings, temporal context, measuring scales, approaches, and aims of the included studies, we have not quantitatively assimilated the findings about dysmenorrhoea prevalence in community settings, but have visually mapped the figures for prevalence in the evidence we identified in Figure 2, Table 3 (references in Supplementary Appendix B). While there is a range of prevalence within and between countries, we note evidence of widespread documentation of symptomatic dysmenorrhoea. This aligns with a systematic review including 21,573 participants aged under 25 years which found an aggregate prevalence of 71.1% with no significant difference between Low and Middle Income Countries (LMIC) and High Income Countries (HIC). This review also reported no significant differences in prevalence related to the age of participants, or whether they were at school or University (77). In England, a 2021 study surveyed 442 secondary school students in Birmingham (a 53% response rate). Strikingly, 93.6% reported experiencing menstrual pain (46.2% reporting pain every month) and 63% believed their periods were normal with 27% unsure if they were normal and 30% unsure if they were regular (81). Papers included in this synthesis assert that 10% of adolescent dysmenorrhoea is secondary (90% primary) (54, 58), however we are unable to identify citations or evidence to substantiate this figure.

**Table 3:** References contributing evidence to this map, detailed in Supplementary Appendix B.

Country	Details, reference numbers refer to references cited in Supplementary Appendix B
Australia	Studies = 3, *N* = 5,607, prevalence range 80%–93%, references 1–3
Belgium	Studies = 1, *N* = 769, prevalence amongst 13 year olds 42%, reference 4
Brazil	Studies = 1, *N* = 105, prevalence dysmenorrhoea = 73%, reference 5
Canada	Studies = 1, *N* = 289, prevalence = 93%, reference 6
China	Studies = 3, *N* = 11,083, prevalence 42%–94%, references 7–9
Croatia	Studies = 1, *N* = 297, prevalence 55%, reference 10
Egypt	Studies = 2, *N* = 949, prevalence 66%–75%, reference 11–12
Ethiopia	Studies = 2, *N* = 1,071, prevalence 65%–72%, references 13,14
Finland	Studies = 2, *N* = 4,473, prevalence 48% (12 year olds), 68%–79% older adolescents, references 15,16
France	Studies = 1, *N* = 4,203, prevalence = 21%, reference 17
Georgia	Studies = 1, *N* = 2,561, prevalence = 52%, reference 18
Ghana	Studies = 1, *N* = 456, prevalence = 74%, reference = 19
Hong Kong	Studies = 2, *N* = 6,262, prevalence = 66%–69%, references 20,21
India	Studies = 8, *N* = 3,976, prevalence 56%–79%, references 22–29
Iran	Studies = 1, *N* = 897, prevalence 68%, reference 30
Israel	Studies = 1, prevalence = 81%, reference 31
Italy	Studies = 1, *N* = 356, prevalence 85%, reference 32
Japan	Studies = 3, *N* = 2,535, prevalence 65%–85%, reference 33–35
Jordan	Studies = 1, *N* = 596, prevalence 38%, reference 36
Korea	Studies = 2, *N* = 1,110, prevalence 74%–82%, reference 37,38
Lebanon	Studies = 1, *N* = 389, prevalence = 74%, reference 39
Malaysia	Studies = 3, *N* = 4,798, prevalence = 68%–76%, reference 40–42
Mexico	Studies = 1, *N* = 1,152, prevalence = 48%, reference 43
Morocco	Studies = 2, *N* = 859, prevalence = 70%–78%, reference 44,45
Nepal	Studies = 1, *N* = 61, prevalence = 79%, reference 46
Nigeria	Studies = 4, *N* = 3,256, prevalence 64%–82%, reference 47–50
Oman	Studies = 1, *N* = 404, prevalence 94%, reference 51
Palestine	Studies = 1, *N* = 956, prevalence 85%, reference 52
Saudi Arabia	Studies = 1, *N* = 344, prevalence 74%, reference 53
Singapore	Studies = 1, *N* = 5,561, prevalence = 82%, reference 54
Sri Lanka	Studies = 1, *N* = 200, prevalence = 84%, reference 55
St Vincent and Grenadine	Studies = 1, *N* = 478, prevalence 45%, reference 56
Sweden	Studies = 3, *N* = 7,772, prevalence 27%–89%, references 57–59
Switzerland	Studies = 1, *N* = 3,340, prevalence 87%, reference 60
Taiwan	Studies = 2, *N* = 1,363, prevalence 73%–85%, reference 61,62
Tanzania	Studies = 1, *N* = 880, prevalence 74%, reference 63
Thailand	Studies = 1, *N* = 789, prevalence 84%, reference 64
Turkey	Studies = 6, *N* = 22,903, prevalence 39%–92%, references 65–70
UK	Studies = 3, *N* = 3,203, prevalence 77%–94%, reference 71–73
USA	Studies = 6, *N* = 20,262, prevalence 60%–85%, references 74–49

### What might contribute to dysmenorrhoea (epidemiological associations)

The relationship between socio-economic status (SES) and dysmenorrhoea is inconsistent, with some finding increased prevalence with increased SES (82, 83), and others no relationship (37, 84, 85). Differing variables to characterise socio-economic status are employed, such as household structure (45, 86) or income (29, 86) and paternal profession (87), which further complicates drawing conclusions. Potential confounders which could complicate understanding these potential associations include access to care and research inclusion. The literature documents other associations, including marital or relationship status (12, 88, 89), religious beliefs (2), family dynamics (45, 86, 87, 90, 91), educational level (90), ethnicity (including being in a minority ethnic group) (29), and perceptions of femininity and self-esteem (45, 46, 87). These are likely contextual and thus potentially confounded.

#### Menstrual associations

Dysmenorrhoea increases in incidence during adolescence with both chronological and gynaecological age (16, 31, 35, 57, 61, 85, 92–96). While the natural history of adolescent dysmenorrhoea is imperfectly understood (97), evidence suggests that rates of dysmenorrhoea peak in mid to late adolescence (2, 94, 98–103), becoming lower in adulthood than adolescence (66, 73, 104).

Early age at menarche is suggested as a risk factor for adolescent dysmenorrhoea (57, 105–107), however the evidence is inconsistent (19, 29, 70, 98). Some of this relationship may be attributable to the positive association between increasing gynaecological age (number of years since menarche) and dysmenorrhoea, with a large 2018 study reporting no association between age at menarche and dysmenorrhoea once gynaecological age was accounted for (94).

We found a range of assertions about the “typical” interval between menarche and the onset of menstrual pain, including what this time interval implies about the likelihood that dysmenorrhoea is primary or secondary. These include that (primary dysmenorrhoea) menstrual pain is variously expected to onset within 6–12 months of menarche (15 studies) (2, 103, 105, 107–118), 6–12 months after menarche (10 studies) (12, 25, 50, 119–125), within 6–24 months (2 studies) (65, 126), within 12–36 months (11 studies) (72, 82, 83, 98, 127–133), or after 12–36 months (several years) (11 studies) (13, 29, 55–58, 73, 102, 134–136). These frequencies are summarised in Table 4. Some authors advise that onset of pain within 6 months is abnormal and should be investigated (65). However, in several primary studies (28, 36, 87, 98, 101), including qualitative accounts (137, 138) many adolescents report that their pain started from their first period.

**Table 4:** Documented rates of missing school or work from references identified in this synthesis, full reference details in Supplementary Appendix C.

References are detailed in the reference list cited in Supplementary Appendix C
Australia: studies = 3, *N* = 5,641, 26%–37% report missing school or work, references 1–3
Brazil: Studies = 1, *N* = 205, 31% report missing school or work, reference 4
Croatia: studies = 1, *N* = 297, 12% report missing school or work, reference 6
Egypt: studies = 1, *N* = 664, 20% report missing school or work, reference 7
Ethiopia: Studies = 1, *N* = 1,071, 31–65% report missing school or work, reference 9,10
Finland: studies = 2, *N* = 4,473, 8%–21% report missing school (and *N* = 1,103: 49.9% with dysmenorrhoea miss school), reference 11,12
Georgia: studies = 1, *N* = 2,561, 70% report missing school or work, reference 13
Hong Kong: studies = 2, *N* = 6,262, 6%–7% report seeing a HCP, reference 14,15
India: Studies = 6, *N* = 2,153, 17%–45% report missing school or work, references 16–21
Jordan: studies = 1, *N* = 596, 8% report missing school or work, reference 22
Lebanon: studies = 1, *N* = 389, 41% report missing school or work, reference 24
Malaysia: studies = 3, *N* = 4,798, 11%–15% report seeing a HCP, references 25–27
Mexico: Studies = 1, *N* = 1,152, 12% report missing school (24% of those with dysmenorrhoea), reference 28
Morocco: studies = 2, *N* = 859, 6%–13% report missing school or work, references 29,30
Nigeria: studies = 1, *N* = 400, 57% report missing school or work, reference 31
Oman: studies = 1, *N* = 404, 45% report missing school or work, reference 32
Palestine: Studies = 1, *N* = 956, 31% report missing school or work, reference 33
Saudi Arabia: Studies = 1, *N* = 344, 51% report missing school or work, reference 34
Singapore: Studies = 1, *N* = 5,561, 24% report missing school or work, reference 35
Sri Lanka: Studies = 1, *N* = 200, 44% report missing school or work, reference 36
Sweden: Studies = 2, *N* = 2,287, 14%–37% report missing school or work, references 37,38
Taiwan: Studies = 1, *N* = 760, 9% report missing school, 34% miss physical training, reference 39
Thailand: Studies = 1, *N* = 789, 21% report missing school or work, reference 40
Turkey: Studies = 4, *N* = 18,495, 16%–28% report missing school or work, references 43–46
UK: Studies = 1, *N* = 442, 23% report missing school or work, reference 47
USA: Studies = 2, *N* = 3,405, 14%–38% report missing school or work, references 48,49

The relationship between menarche and onset (and causation) of primary dysmenorrhoea is frequently related to the onset of (regular) ovulatory cycles (13, 57, 58, 82, 117). However, recent evidence sheds doubt on this, with studies showing that anovulatory cycles are commonly painful (47, 131), irregular cycles are painful (139), that painful cycles are significantly more frequent than ovulatory ones (140), and that regular cycles do not reliably predict ovulation.

Studies differ as to whether heavy (37, 141) or longer duration (98) menstrual flow increases the likelihood of dysmenorrhoea (98), or alters the timing of pain relative to bleeding (139), although most suggest that pain is more likely with heavier flow and longer cycles (2, 28, 31, 85, 101, 104, 107, 142, 143). The relationship between pain and irregular cycles is inconsistent, with some studies finding an association between irregular cycles and pain (14, 16, 29, 32, 114, 144), and others no relationship (19, 33, 37, 106, 145). Irregular cycles can cause heavier unpredictable bleeds, which complicates this relationship (106).

There is a consistently documented relationship between pre-menstrual syndrome (PMS) or pre-menstrual dysphoric disorder (PMDD) and dysmenorrhoea (59, 92, 108, 146–148). This may be reciprocal, with PMS exacerbating pain and pain increasing the likelihood of PMS (106, 149).

A family history of menstrual pain is associated with an increased likelihood of dysmenorrhoea (30, 64, 83, 101, 150–153). This was historically attributed to maternal factors (anxiety, role modelling or dominance) (41, 45, 47, 62, 67, 83, 154) however a study comparing adolescents, their peers and their mothers found that adolescents experience significantly more symptoms than their mothers, and were less likely to view menstruation as a “positive event” (155). A family history of endometriosis also increases the likelihood of endometriosis (66, 156).

#### Non-menstrual associations

The relationship between dysmenorrhoea and BMI is inconsistent, with some studies reporting increased dysmenorrhoea in those with low (<16.5) (29, 35, 72, 157, 158) or raised BMIs (143, 158), while others not identifying a relationship (2, 13, 19, 98, 104, 113, 159, 160). This relationship may be complicated because being taller and thinner is also an identified epidemiological risk factor for endometriosis (156). Regardless of BMI, some authors report increased rates of dysmenorrhoea in young people who have dieted to lose weight (73, 158, 161, 162). Separate work finds that dysmenorrhoea is associated with an increased likelihood of “body image dissatisfaction” (163) or “negative self-perception” (164). Some authors relate the likelihood of dysmenorrhoea to eating junk food (110, 159). There are papers reporting an association between irregular meals or skipping breakfast with dysmenorrhoea (29, 165–167). While the authors conclude that the act of skipping meals may drive pain (165–167), we note that nausea (14, 18) and altered appetite (21, 24) are well documented symptoms associated with dysmenorrhoea which might cause young people to miss meals.

Two studies report that those with dysmenorrhoea drink more caffeine (62, 110), postulating the vasoconstrictor action of caffeine as a potential mechanism (110). In parallel, large observational studies demonstrate that dysmenorrhoea significantly adversely impacts quality and quantity of sleep, and is associated with significant daytime sleepiness (148, 168, 169). Reducing caffeine is used by some adolescents as a self-care strategy for dysmenorrhoea (170), while caffeine is a component of some medications used for dysmenorrhoea (171). Qualitative accounts demonstrate that dysmenorrhoea's impacts on sleep disruption are followed by fatigue (172), which could potentially contribute to the observed relationship between caffeine consumption and pain.

Evidence consistently links cigarette smoking with an increased incidence of dysmenorrhoea in teenagers (85, 104, 173–175). The associations between dysmenorrhoea and alcohol drinking in adolescence is less clear, with inconsistent findings about whether dysmenorrhoea is reduced or aggravated by alcohol (85, 143) and some find reducing alcohol effective in reducing pain (170). For both smoking and alcohol, the risk of social acceptability bias in self-reported evidence could under or overestimate the strength of these associations.

The relationship between exercise and the likelihood of dysmenorrhoea has been inconsistent (13, 104, 113, 141, 143, 145, 176–178). However, now two publications by Cochrane suggest that both high and low intensity exercise can alleviate menstrual pain, notably in young women, although whether this persists after exercise stops or impacts on quality of life is unknown (179, 180).

Historically, specific exercise programmes have been advocated as therapeutic interventions to treat or prevent menstrual pain (181–185). A trial studying yoga suggested therapeutic benefits for dysmenorrhoea (186). Both avoiding and doing exercise are commonly reported effective self-care strategies by adolescents with dysmenorrhoea. As well as impacts on fatigue and pain, potential contributory factors that could contribute to this include beliefs that people should avoid exercise whilst menstruating, or concerns about flow and leakage. In a survey study, Campbell showed that 90% of teenagers use resting to manage pain (with 60% finding this effective), while in the same study, 57% actively used exercise to relieve pain, (and 52% felt it worked) (170). Qualitative accounts similarly describe avoiding (138, 187, 188) and using exercise to manage menstrual pain (116). Dusek reports lower rates of dysmenorrhoea in elite athletes, compared with controls, however a third of elite athletes had amenorrhoea, reducing the number of painful cycles, which was not accounted for (189).

#### What conditions may be associated with dysmenorrhoea?

The impact of menstrual pain on emotional and psychological wellbeing is demonstrated in research, with dysmenorrhoea increasing the number and severity of depressive symptoms (90, 163, 168, 190, 191) and depression symptoms increasing menstrual symptoms (93) There is no consistent association between dysmenorrhoea and personality or psychosocial variables (46, 52, 66, 76). Pain is associated with (and can contribute to) negative expectations of menstruation (37, 192) and negative expectations of menstruation impact on menstrual experience (49, 193) Not accepting menstruation alongside holding stronger beliefs in external health locus of control was shown to predict seeking medical help (194). Dysmenorrhoea is related to lower perceptions of self-efficacy, with the level of pain predicting menstrual distress (91) and the extent to which this manifests as reduced engagement with activity. High levels of pain catastrophizing accompany higher levels of menstrual pain (195, 196), and flexible coping strategies may help mitigate against menstrual pain (197, 198). It has been suggested that the realisation that the onset of menstruation implies a recurring experience of dysmenorrhoea potentially exacerbates adolescent distress (49, 192), however others demonstrate that experience and habituation can diminish menstrual distress (73).

Menstrual pain increases stress (101, 150) and stress can aggravate menstrual pain (14, 86). For example, annual surveys in Japan reported increased dysmenorrhoea after the 2011 earthquake and tsunami (199). There is a reported relationship between childhood stressors and dysmenorrhoea, including sexual abuse (122) and conflicted domestic relationships (90, 112).

Research with young people with autism or cerebral palsy documents increases in difficult or distressed behaviours preceding and during the menstrual period (200–202), which can be helped by offering treatment for menstrual pain (200) (including hormonal treatments such as the intra-uterine system) (203).

We identified one paper associating ADHD symptoms (not diagnosis) from a screening tool (T-DSM-IV-S) in young people recruited from a child and adolescent service, which identified correlation between symptoms of dysmenorrhoea and inattention and impulsivity, impaired sleep and mood impacts (164).

An emergency room (uncontrolled) case series suggests a possible association between dysmenorrhoea, described as not responsive to non-steroidal anti-inflammatory medications (NSAIDs), and Familial Mediterranean Fever (FMF), noting that acute FMF pain episodes can triggered by the menstrual cycle (204).

Dysmenorrhoea is associated with other pain conditions [including chronic pelvic pain, IBS, fibromyalgia, temporomandibular joint (TMJ) pain, headaches and musculoskeletal pain] (205).

Dysmenorrhoea is associated with non-cyclical pain and chronic pelvic pain (47, 206). This relationship is complex, because pelvic pain may be a marker of adolescent endometriosis (207), as can dysmenorrhoea (208). However, in a large (1,785 adolescents) web survey in 2017, 44% of respondents reported acyclic pelvic pain (34). A correlation between irritable bowel syndrome (IBS) and both primary dysmenorrhoea and PMS has been reported (209).

Another condition linked with dysmenorrhoea is headache, especially menstrual and peri-menstrual headache (210, 211). Headache frequency increases with gynaecological age. Headaches are more common in combined hormonal contraception (CHC) users (210), (although headache as a side effect of CHC use may contribute to this observation).

In a systematic review, having dysmenorrhoea was associated with 2.5 times the odds of living with another chronic pain condition. While not exclusively focussed on adolescents, the majority of studies included adolescents, and three included only adolescents (reporting on IBS pain, TMJ pain, and migraine/headache). The authors reflected on the potential significance of adolescence as a time of neurodevelopmental plasticity as it relates to pain (205). Dysmenorrhoea is known to cause central pain sensitisation (205).

## What impacts does dysmenorrhoea have on adolescents?

### Education and work

**Figure 3:**
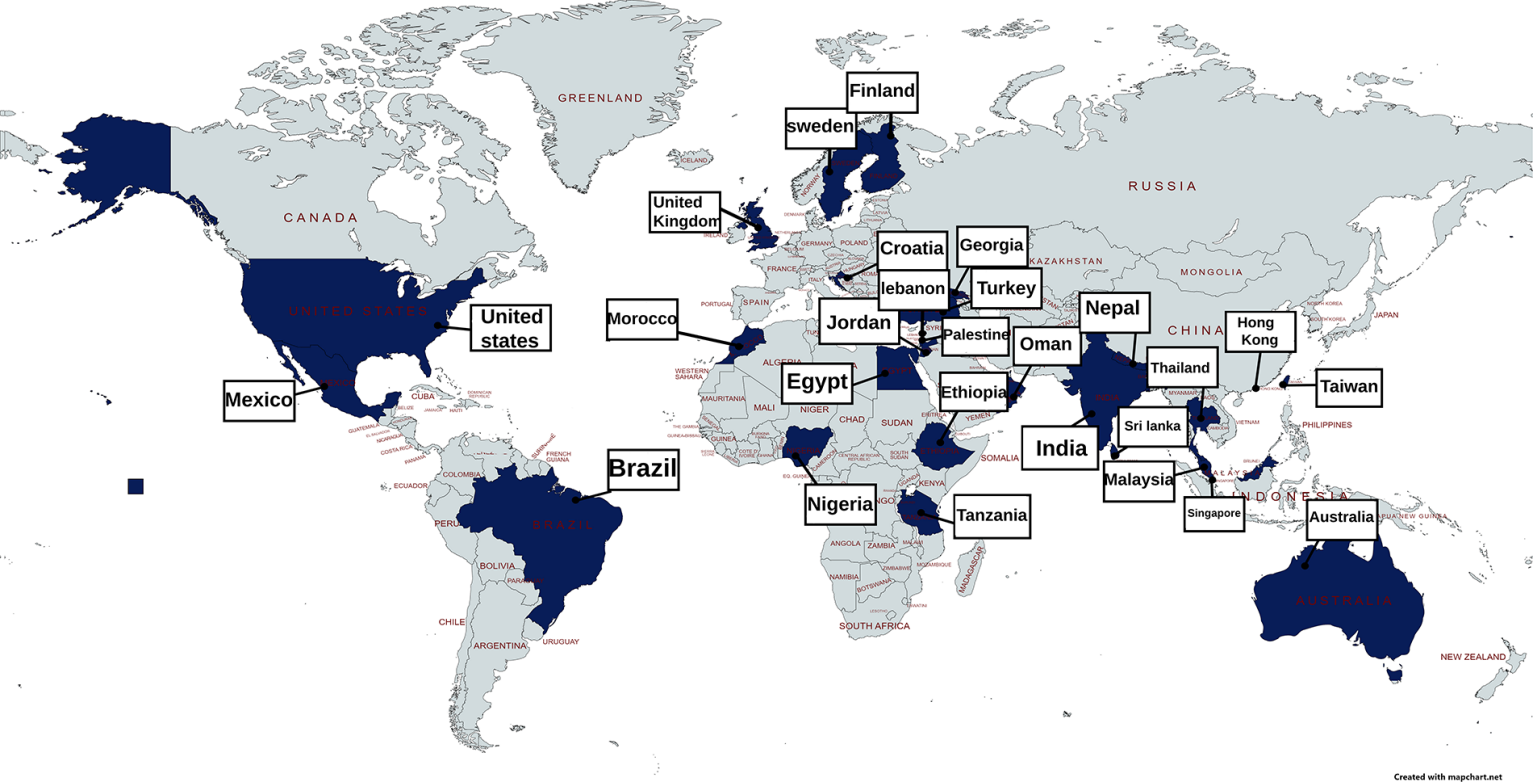
Visual mapping of documented figures for missed school or work because of adolescent dysmenorrhoea.

A systematic review of educational impacts of dysmenorrhoea including data from 11,226 young women aged <25 years in 19 studies found that 20.1% had missed school or university because of dysmenorrhoea and for 40.9%, dysmenorrhoea adversely affected academic work and classroom performance (77). This aligns with the studies included within this review, demonstrating a similar range of school or work absenteeism from 6%–70% in papers that reported this. The papers that differentiated school absence rates between mild, moderate, and severe dysmenorrhoea showed that absence rates tend to be higher for those who report more severe pain. There were adverse impacts reported on concentration, test performance and being able to study or do homework. This is summarised in Figure 3 and Table 4 (Reference list is cited in Supplementary Appendix C).

In a 2021 survey of 442 English school girls, 22.7% sometimes missed school because of their periods. Most absences were attributed to pain, but for 25% managing heavy bleeding contributed (81). The interface between bleeding and school attendance is illustrated in Li's qualitative study which highlights challenges experienced by school students in accessing pain relief and products to manage menstrual flow in school alongside negotiating necessary trips to the toilet (79). The potential contribution of menstrual poverty adds to the challenges of managing menstruation in school and work.

### Social and leisure activities

Menstrual pain reduces young people's engagement with leisure activities, including sports and hobbies (188), and socialising with peers and families (137, 172). It can impact on social interactions (172, 187) including sexual activity (212).

### Well-being and quality of life

The experience of adolescent dysmenorrhoea was associated with a reduction in health-related quality of life in two studies, one of which found a reduction in the physical score domain (213), and one of which found reductions in both physical and social domains (115).

### Long-term outcomes

The lack of evidence describing the natural history of adolescent dysmenorrhoea is a documented research need (97). This includes whether adolescent dysmenorrhoea predicts health events or conditions in later life (including endometriosis, sub-fertility, pelvic pain or other pain conditions) and also whether interventions in adolescence influence any of these potential outcomes (214, 215). However, a small 1983 cohort study reported that irregular or painful periods in adolescence predict poorer gynaecological health in adulthood (42).

Longitudinal and cohort studies suggest a trend towards a reduction in severity and incidence of menstrual pain after adolescence. We identified one cohort study following up 148 adolescents seen in an Australian specialist tertiary care clinic for (presumed severe) dysmenorrhoea ten years later. Of the 70 who could be contacted, 30.4% had ongoing severe pain, but 27.1% had no, or slight, pain with menstruation, representing significant improvement. They did not identify characteristics in adolescence that predicted enduring severe menstrual pain (97).

## What do we know about how and when adolescents access information and care for dysmenorrhoea?

### Accessing information

Most young people learn about periods from their mothers, or other family members (20, 23, 38, 80, 100, 121, 216), followed by friends and teachers (26, 114). Only a minority receive menstrual information in healthcare settings (71, 80, 193). Education and knowledge improve self-care agency (217, 218), support effective use of treatments (123, 219), and increase awareness of endometriosis and attendance for healthcare (220). Information and education help young people feel prepared for menarche; encountering menarche without knowledge can be frightening and difficult (74, 138, 221).

Advertising is another potential source of menstrual information. A 1988 thematic analysis of menstrual product advertisements in a magazine targeted for adolescents concluded that menstrual adverts depict menstruation as a “hygiene crisis” requiring technology and science to ensure that leaks and odour are contained, while maintaining secrecy (and menstrual stigma). Menstruating people were characterised as full of energy, often wearing white and not allowing menstruation to hinder their activity or lifestyle, in stark contrast to the accounts of the impacts of menstrual pain on many young people (78).

In what is likely to be an evolving practice, adolescents reported using the internet to find information about menstruation (75). In a big data exploration mapping 1.9 billion enquiries made to a chat and answer platform in the USA in 2018, 84% of queries about menstruation were from adolescents, suggesting a significant unmet information need. Questions clustered around themes such as managing menses in school, treatment and self-care options and about possible underlying causes or consequences of menstrual pain, although queries about specific causes of dysmenorrhoea including endometriosis were rare (0.05% of female queries) (222). This aligns with the lack of knowledge and awareness of endometriosis, alongside a desire to know more about it, depicted by Randhawa et al. among English secondary school students (81). A UK study from 2011 evaluated the content of internet information about dysmenorrhoea for adolescents. Of the 23 websites they identified which advised on dysmenorrhoea, only one was targeted at adolescents. Although 65% of dysmenorrhoea sites advised seeking health professional advice, the paper concluded that, in 2011, the internet sites were of low quality and uncertain value in supporting adolescents experiencing pain (223).

### Accessing health services

**Figure 4:**
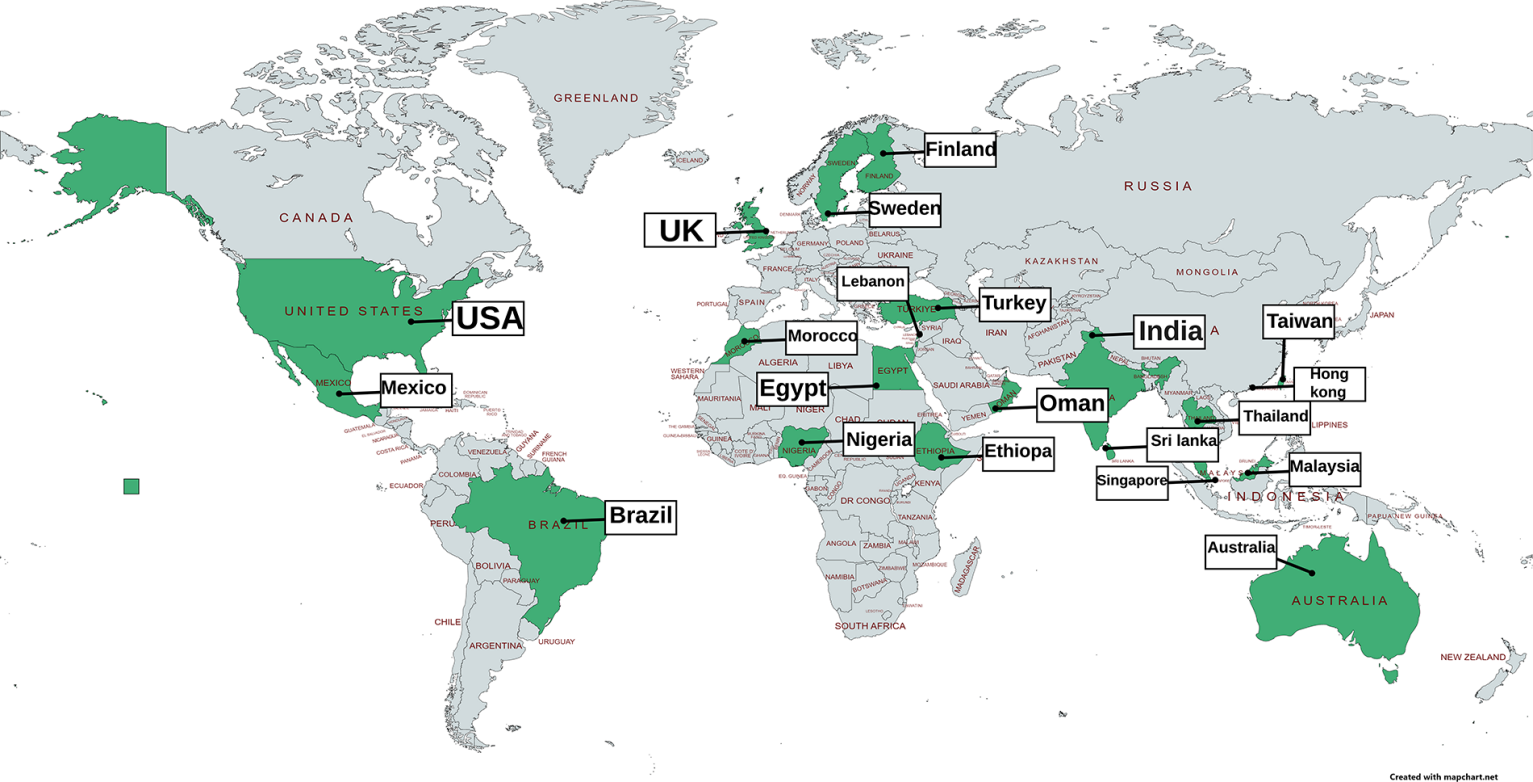
Visual mapping of documented figures of the proportion accessing healthcare services because of adolescent dysmenorrhoea.

The majority of young people with dysmenorrhoea do not seek medical help, with a systematic review reporting an aggregate figure of 11% seeking healthcare which did not significantly differ between high or low and middle income countries (224). Increasing pain predicts increasing attendance at healthcare (31, 194). In the UK, Randhawa's study found that 29.5% of young women had seen a GP (81), although little is known about what happens when they do. In the studies in this synthesis, we found that rates of having seen a doctor or nurse, varied between 0% and 34%. There are country specific factors which influence accessing healthcare, for example studies in China report young people choosing between traditional and Western practitioners (188), and in some health systems the cost of accessing healthcare is a deterrent. Figure 4 and Table 5 map documented attendance at healthcare settings (reference list is cited in Supplementary Appendix C).

**Table 5:** Documented rates of attendance at healthcare settings for adolescent dysmenorrhoea from references identified in this synthesis, full reference details in Supplementary Appendix C.

Reference list for these studies is detailed in Supplementary Appendix C
Australia: studies = 2, *N* = 1,439, 18%–33% report seeing a HCP, references 1,2
Brazil: Studies = 1, *N* = 205, 28% report seeing a HCP, reference 4
Canada: Studies = 1, *N* = 289, 18% received prescribed pain medication, reference 5
Egypt: studies = 2, *N* = 949, 3%–13% report seeing a HCP, references 7,8
Ethiopia: studies = 1, *N* = 612, 11% report seeing a HCP, reference 10
Finland: Studies = 1, *N* = 1,103. 49 had severe dysmenorrhoea and 53% of these had seen a HCP, reference 12
Hong Kong: studies = 2, *N* = 6,262, 12%–16% report missing school or work, reference 14,15
India: studies = 3, *N* = 722, 4%–11% report seeing a HCP, references 18–20
Korea: studies = 1, *N* = 538, 0.1% Western HCP, 0.4% Oriental practitioner, reference 23
Lebanon: studies = 1, *N* = 389, 7% report seeing a HCP, reference 24
Malaysia: studies = 3, *N* = 4,798, 11%–15% report seeing a HCP, references 25–27
Mexico: Studies = 1, *N* = 1,152, 13% had seen a HCP (28% of those with dysmenorrhoea), reference 28
Morocco: studies = 2, *N* = 859, 6%–10% report seeing a HCP, references 29,30
Nigeria: studies = 1, *N* = 400, 0% reported seeing a HCP, reference 31
Oman: studies = 1, *N* = 404, 3% report seeing a HCP, reference 32
Palestine: Studies = 1, *N* = 956, 0% report seeing a HCP, reference 33
Saudi Arabia: Studies = 1, *N* = 344, 18% report seeing a HCP, reference 34
Singapore: Studies = 1, *N* = 5,561, 13% report seeing a HCP, reference 35
Sri Lanka: Studies = 1, *N* = 200, 13% report seeing a HCP, reference 36
Sweden: Studies = 1, *N* = 1,785, 7% report seeing a Dr, 33% seeing school nurse, reference 37
Taiwan: Studies = 1, *N* = 760, 21% report seeing a HCP, reference 39
Thailand: Studies = 1, *N* = 789, 7% report seeing a HCP, reference 40
Turkey: Studies = 5, *N* = 20,952, 1%–10% reported having seen a HCP, references 41, 42, 44–46
UK: Studies = 1, *N* = 442, 30% report seeing a HCP, reference 47
USA: Studies = 1, *N* = 706, 14% reported seeing a HCP/Dr, 49% had seen the school nurse, reference 48

Believing pain is inevitable or normal can deter people from seeking medical advice for menstrual pain (2, 71, 73, 123, 142, 224, 225). Young people may become habituated to pain with time, describing this as better (73). Where there are differentials in attendance reported by health setting, higher numbers seek care from school nurses than doctors (20, 34).

Documented barriers to accessing healthcare include previous adverse experiences of healthcare for dysmenorrhoea (188), fears of not being taken seriously (109) or of the pain or menstrual problems being normal (74, 226), and worry about “embarrassing” questions or being examined (22, 95).

Menstrual stigma and etiquette remain barriers to accessing advice and help (80, 226). This is reflected in the number of apologies for asking “embarrassing” questions on the anonymous chat assessment in an analysis of 1.9 billion menstruation queries on a USA question and answer internet platform (ChaCha) (222), and is also seen in the language young people use to describe their periods (e.g., one survey with free text box responses reported use of the words “disgusting”, “ridiculous” and “embarrassing”) (37).

Menstrual education has been shown to increase attendance at healthcare (220).

## When might adolescent dysmenorrhoea signal the presence of a contributory cause?

### Congenital developmental malformations

While developmental anomalies can present in a variety of ways and at any age, adolescence is a not uncommon time for identification and diagnosis of these, if they are associated with menstrual symptoms which develop following menarche (227–229). While some anomalies result in the absence of menarche, in this paper we consider developmental anomalies that may present with period pain in adolescence. For some anomalies, severe dysmenorrhoea was a prominent presenting symptom. For example, in their review of the literature about cystic adenomyosis, Brosens et al. identified 21 case reports, 18 of which presented with dysmenorrhoea (227). A number of case reports document presentation with pain which localises predominantly to one side (unilateral dysmenorrhoea) (230–232).

Pain onset is often marked and worsening from menarche (233–244), although some case reports document onset after menarche (245–250). This may depend on the type of anomaly, including for example whether there is obstruction to menstrual flow (251, 252). There is a wide range of anomalies that can present with dysmenorrhoea including uterine didelphys (252–255), those termed accessory cavitating uterine mass (230, 233, 234, 250), Roberts Uterus (228), Herlyn–Werner–Wunderlich syndrome (obstructing hemivaginal septum, uterus didelphys, and ipsilateral renal agenesis) (238, 244, 251), rudimentary horns (229, 231, 232, 237, 239, 243, 245, 256), and unicornuate uterus (Mullerian anomalies) (236, 253). These can co-exist and evaluating congenital anomalies requires specialist input and imaging (for example MRI or HSG), and treatment (227).

Pelvic anomalies can be associated with other developmental anomalies, especially of the genitourinary and renal tract (245, 251–253). Where ultrasound findings were documented in the case report, they reported anomalies that would clearly indicate a need for referral from primary to secondary care for specialist assessment (232, 235, 236, 239–242, 249, 250, 256–259). Congenital structural anomalies also increase the likelihood of endometriosis (118, 232, 251, 253, 260).

### Endometriosis

Endometriosis is a chronic inflammatory condition defined as the presence of endometrium like tissue outside of the uterus (66, 261). Endometriosis used to be considered rare in adolescents (262–264), but does occur: the majority of adult women with endometriosis report symptom onset in adolescents (265, 266). Like adult women, adolescents experience significant delays in diagnosis (262, 267).

There is uncertainty about the community prevalence of adolescent endometriosis (156, 214), in part because historically diagnosis required a laparoscopy (266), which could only be undertaken in specialist settings (214). Rates of endometriosis confirmed in the population of adolescents who are investigated with laparoscopy or imaging for chronic pelvic pain or dysmenorrhoea are strikingly high, with systematic reviews citing retrospective rates of 62% (207, 268) in adolescents with chronic pelvic pain, in 75% in those with chronic pelvic pain resistant to medical treatment (and 49% in others with chronic pelvic pain) and in 70% with (severe) dysmenorrhoea (268). However, the community prevalence of endometriosis is unknown (214), raising a significant denominator question (214, 265, 269). Adult women are often diagnosed with endometriosis through either a pain or fertility route, whilst adolescents tend to be diagnosed only when significantly symptomatic, which influences prevalence (214). In addition, the symptoms of those diagnosed with endometriosis in adolescence may differ from those diagnosed in adulthood, with a cross-sectional analysis of a longitudinal cohort identifying adolescents report more pain from menarche, more acyclic pelvic pain and more nausea (265). Across the cohort, 90% had dysmenorrhoea (265). Trans-masculine people experience endometriosis and the intersection between this and hormonal interventions in an area where greater understanding is needed (269).

Guidance and expert opinion pieces suggest referring any adolescent whose pain has not improved within 3–6 months of empirical therapy with NSAIDs and/or hormonal treatment for consideration of possible endometriosis (13, 65, 66, 109). We were unable to identify evidence about further discriminatory assessments for adolescents whose pain *was* resolved (or improved).

The interface between symptoms and response to empiric treatment is pivotal in guidance about referral for further investigation for endometriosis (109). The relationship between combined hormonal contraception and endometriosis is complex (156). Hormonal contraceptive medication is an evidence-based treatment for both dysmenorrhoea and endometriosis-associated pain (117). Whether these treatments influence the underlying processes of endometriosis, either to reduce progression or scarring and potential future sequelae, or contribute to disease progression directly whilst suppressing (or “masking”) symptoms is uncertain (117, 118). A systematic review (including predominantly adult women) reported a reduced risk of endometriosis diagnosis in current CHC users (OR 0.63) and an increased risk in past users (OR 1.21). The authors note the possibility that symptom suppression makes referral for investigation less likely (117) (and we note is embedded in clinical guidance) (3, 270). It is possible that adolescent CHC use is a marker for severe dysmenorrhoea and thus a confounder not cause of epidemiological associations between CHC use and endometriosis. A retrospective case series of 410 women found that previous CHC use for severe “primary” dysmenorrhoea was associated with an increased risk of a diagnosis of deep infiltrating endometriosis (OR 5.6) and other endometriosis (OR 2.6) in adulthood. They also note that this may be because CHC use for dysmenorrhoea functions as a marker for endometriosis and not necessarily a contributory cause (271).

The evidence associating non-response to hormonal contraception therapy as a marker of likely endometriosis is inconsistent. In retrospective case series, where the cases have a surgically confirmed diagnosis of endometriosis, many had dysmenorrhoea that was refractory to medical treatment (268). But, a prospective case series following young people with marked dysmenorrhoea seen in a specialist clinic, 92.2% achieved positive symptomatic responses to hormonal therapy, NSAIDs or tranexamic acid. Of the 8.8% (*N* = 2/16) of adolescents who had a laparoscopy, 2 had endometriosis (139). Knox followed up participants ten years after they were seen in tertiary care with adolescent dysmenorrhoea and found that using AND having a positive therapeutic response to CHC in adolescence (rather than a lack of response) predicted adult endometriosis (97). A prospective case series in Egypt found that of the 654 adolescents they interviewed, 48.9% had dysmenorrhoea and of these 68.8% reported severe dysmenorrhoea. Of the 320 adolescents they studied with dysmenorrhoea, 100 responded to medical therapy and were then not assessed further. Of the 220 whose pain did not resolve, 56 had USS findings suggestive of endometriosis and 34 of these agreed to laparoscopy (22 declined), and 27/34 (12%) had endometriosis. Those with a negative ultrasound scan were not investigated further in this study (272). In a 2013 systematic review, the authors report the “unexpected” finding that the prevalence of moderate or severe endometriosis was lower in girls with chronic pelvic pain or dysmenorrhoea resistant to CHC or NSAID treatment, when compared with a group with CPP not resistant to treatment, speculating that treatment may not prevent development of endometriosis, but may limit progression to severe disease (268).

In these studies, threshold for laparoscopy is a critical determinant of what we understand about endometriosis prevalence, and the interface between symptoms and response to treatment is an important component of determining this threshold. However, in a real-world case series of adolescents (aged 12–20) presenting to an out-patient ultrasound clinic between 2014 and 2019, ultrasound signs of endometriosis were found in 21% of the 147 adolescents referred with dysmenorrhoea, rising to 33% who also reported dyspareunia. It is worth noting that any young people taking hormonal medication were excluded from this study (273).

Once a diagnosis is made, there is evidence for surgery and hormonal therapy to reduce recurrence (208). There is a risk of recurrence after surgery, which may be higher in adolescence than in adulthood (274), which is a significant concern associated with risks of repeated surgeries and pain (215). Specialist treatment can also include menstrual suppression with Gn-RH analogues, possibly with add back hormone replacement therapy (208). This should be initiated within specialist care settings, who can advise on follow up, monitoring and treatment duration. However, general practice teams may be involved in supporting this process, for example administering treatment or prescriptions.

In general practice, awareness of the diagnosis of endometriosis can facilitate recognition and response to potential symptomatic recurrence and supports shared decision making (66). Recognising that endometriosis is a chronic and complex condition, alongside physical treatments, there is a need for ongoing educational, emotional and psychological support (275).

### Adenomyosis

Adenomyosis is infiltration of the myometrium by endometrial tissue. Although currently considered rare in adolescence, there are case reports linking adolescent dysmenorrhoea to diagnoses of adenomyosis (276, 277).

### Other causes of secondary dysmenorrhoea

The papers included in this review did not offer detail about other causes of secondary dysmenorrhoea beyond a descriptive listing, which includes sexually transmitted infections, pelvic inflammatory disease [which can cause dysmenorrhoea by increasing inflammatory markers and causing adhesions (278), and can present acutely with worsened dysmenorrhoea (279)], pelvic adhesions, and ovarian cysts, and uterine fibroids (109).

## What treatments might help?

### Self-care

Self-care strategies are commonly employed by adolescents with menstrual pain. Self-care encompasses a wide range of potential actions, including physical interventions (exercise or rest) (116, 170, 172, 224), reflective interventions (prayer, distraction, meditation) (170, 172), dietary (food selection or avoidance) (170, 187, 188), pharmacological (herbal, complementary and alternative, OTC analgesia) (116, 171, 172, 187, 224, 225) and non-pharmacological (bathing, avoiding bathing, heat, cold) (17, 152, 170, 188, 225). Self-care strategies, were more likely to be adopted by young people with more severe period pain, by individuals with greater knowledge about dysmenorrhoea or who believed that self-care would be effective (120). Interventions that increase knowledge, such as education in schools, increase self-care behaviour (217, 280).

Many young people use over-the-counter remedies, such as paracetamol or ibuprofen (224, 225), albeit not always taken optimally (171). While offering the potential for self-efficacy, the potential risks of self-medication with inadequate support or knowledge can include harms from choice of painkiller (for example choosing aspirin which is contra-indicated in children), incorrect dosages, incorrect dose intervals, failing to recognise adverse reactions or not seeking healthcare advice when symptoms do not resolve (171, 225). Incomplete or inaccurate understandings of the causes of dysmenorrhoea may align with inappropriate selection of over the counter treatments for dysmenorrhoea (281). Qualitative accounts describe apprehension about side effects, safety and long-term health risks of taking medication including dependence (188), alongside concerns that medication use is unnatural (116, 172, 187). A systematic review showed that the medication most commonly used worldwide for dysmenorrhoea was paracetamol ahead of non-steroidal anti-inflammatory medications for which there is more evidence of benefit in dysmenorrhoea (224).

### Non-pharmacological

Non-pharmacological treatments used by adolescents include physical strategies (locally applied heat, massage, rest, and exercise) (187, 224, 282) and cognitive strategies (distraction, visualisation, seeking support and talking about pain, and keeping busy) (116, 188, 280, 282). In a study comparing methods used alongside perceived effectiveness, physical strategies were experienced as more effective, while psychologically focussed strategies enabled comfort and control (170). It is possible that the degree of pain influences the approaches selected, with more physical and non-pharmacological approaches used by adolescents experiencing more severe menstrual pain (170). Specific interventions with reported benefit include TENS machines (283, 284), external heat application (178, 284, 285), and yoga (186, 284). Systematic reviews evaluating the effectiveness of acupuncture for primary dysmenorrhoea do not consistently identify benefit (133), some noting that the conclusions are limited by methodological flaws in the contributing studies (133, 286), including finding no clear evidence of benefit including when compared to sham acupuncture (286), although reported possible benefits when compared with pharmacological treatment or no treatment (286, 287).

Small studies have explored the benefits of structured psychological therapies on menstrual pain in teenagers and dysmenorrhoea support programmes incorporating CBT methods (218) and relaxation therapy (288) have shown promise, in small un-blinded studies. A Cochrane review included five RCTs of behavioural interventions for dysmenorrhoea, including two trials where the participants mean age was in adolescence, and concluded that these showed promise in reducing pain, albeit with limited evidence because of small numbers of participants and methodological flaws (289).

### Pharmacological

Medications with an evidence base for treating adolescent dysmenorrhoea can be hormonal or non-hormonal. The non-hormonal medications trialled and shown to be effective in teenagers include non-steroidal anti-inflammatory medications (50, 68, 127, 290). There is physiological plausibility for the effectiveness of these medications, which act on prostaglandin pathways implicated in menstrual pain (55). Evidence suggests that NSAIDs are more effective than paracetamol (acetometnophen). Medications trialled and found to be ineffective include beta agonists (reviewed in a Cochrane review) (124) and montelukast (in an RCT including 22 adolescents) (291).

Combined hormonal contraception (CHC) medications reduce pain (292) [including reducing missed school (292) and associated symptoms of dysmenorrhoea such as back pain or nausea] (293). The combined hormonal contraceptive pill reduces prostaglandin levels associated with an improvement in clinical symptoms (56). Analgesic effectiveness has been shown across a range of formulations of CHC (134, 292), including low dose 20 mcg tablets (294). Combined contraception is available in non-oral formulations, including the vaginal ring, which has been shown to be acceptable to young people and effective in reducing dysmenorrhoea (295). Mitigating against dysmenorrhoea is identified by adolescents as a reason to use hormonal contraception even when they do not need contraception (296). When these pills are used primarily for contraception, they may be better accepted when their use is accompanied by non-contraceptive benefits including more tolerable period pain (297–299). There may be a therapeutic benefit from administering the CHC continually, rather than giving it cyclically, and this may be associated with less irregular bleeding (292). We observe that reducing bleeding frequency will by definition reduce dysmenorrhoea, which is pain associated with bleeding, however pragmatically, whilst there is uncertainty about whether pain would persist if bleeding did, this represents a reduction in pain and painful episodes.

Single agent progestogen hormonal contraceptives such as oral desogestrel or the subdermal implant (etonogestrel) are also suggested as potential treatment options for adolescent dysmenorrhoea (65). We identified limited evidence about the effectiveness of oral or subdermal progestogen-only methods in adolescence. A small open label trial of drospirenone in adolescents exploring safety and tolerability documented a reduction in dysmenorrhoea from baseline (300). Observational evidence suggests benefit (including reduced dysmenorrhoea and menstrual suppression) from oral desogestrel (278) and the progestogen-only injection (Depo-provera, DMPA) (55).

The IUS has been shown to be effective (in one case series of 48 teenagers not helped by other interventions 93% reported improved menstrual symptoms) and well tolerated (4% asked for removal within four months of insertion) (301). The IUS was also found to be effective and tolerable in a case series of 14 young people with medical disorders, learning or physical disabilities whose menstrual pain had not resolved with other interventions (203). Young people may experience (301) post-insertional pain after the IUS is fitted, so counselling about this is important.

A number of dietary supplements have been suggested and trialled for adolescent dysmenorrhoea, including zinc (302), Thiamine (vitamin B1) (69, 128), fish oil capsules (omega-3) (128, 303) and vita min E (129, 304). While some small trials have showed potentially beneficial findings, a Cochrane review in 2016 judged all of the available evidence to be of low or very low quality. They reported no evidence for vitamin E. However there was limited evidence for possible benefits for fish oil, Vitamin B1, and zinc, which warrant further research (125). A non-blinded non placebo controlled observational study in Iran treated adolescents (95% had vitamin D deficiency at baseline) with high dose vitamin D and reported improvements in PMS and menstrual pain symptoms (305).

## Uncertainties

### Tensions and inconsistencies

A critical uncertainty that arises from this synthesis is how to characterise the likelihood that pain represents primary dysmenorrhoea rather than secondary. We identify inconsistencies in how primary dysmenorrhoea is described or characterised in research (Table 6).

**Table 6:** Summarises the approaches taken by studies reporting evidence about primary dysmenorrhoea (references refer to reference list cited in Supplementary Appendix D).

Primary dysmenorrhoea ascertained by		Number of studies	Number of participants	Reference numbers (Supplementary Appendix D)
No details cited in method		9	3,652	D2,3,26,38,50,69,70,75,76
Systematic reviews about primary dysmenorrhoea		6	15,266	D49,60,61,62,63,81
Declared assumption (age)		2	766	D 9,24
By excluding secondary dysmenorrhoea	Participant self-report	37	15,766	D4,6,7,8,10,12,14,15,21,22,23,27,28, 30,31,34,36,37,39,41,42,43,44,46, 48,51,53,57,58,59,64,65,66,71,73,78, 82
	Health professional interview (medical history)	1	100	D55
	Health professional assessment including examination (without USS)	5	342	D5,11,16,32,72
	History/self-report and pelvic USS	7	1,336	D29,33,35,54,56,68,80
	Assessment including history, examination, USS and laboratory markers (including Ca-125)	5	3,161	D25,40,45,52, 67
	Negative laparoscopy	1	1	D13
Total number of studies/participants included		73	40,390	
Additional clinical features reported to support study inclusion				
Inclusion features	Timing of onset of symptoms in relation to menarche	3 (1 = symptoms from onset menarche, 1 is symptoms start within 10 months of menarche, 1 symptoms onset 2–3 years after menarche)		D4, 73,80
	Characteristic pain features (“typical” symptoms)	12		D7,11,14,29,40,54,56,59,65,67,71,80
	Regular menstrual cycles	15		D10,11,12,15,37,41,46,51,53,54,56,57,71,73,80
	Un-married/single participants	3		D11,40,51
	Virginity	3		D33,35,56
Exclusion features	IUD *in situ*	12		D4,6,8,31,32,36,37,48, 57,65,66,78
	Taking hormonal contraception	12		D4,8,10,12,32,33,40,41,44,48,56,73
	nulliparous	6		D10,11,25,53,68,73

Apart from laparoscopy, none of these approaches will reliably exclude all causes of secondary dysmenorrhoea and therefore studies that define and delineate expectations of primary dysmenorrhoea likely include participants with both primary and secondary dysmenorrhoea. These research observations contribute to defining the expected characteristics of primary dysmenorrhoea, which are in turn embedded in clinical guidance (54, 89, 109). Examples include the inconsistent reporting of the association between ovulation and pain, age at menarche and pain, and age and pain, and how these relate to the likelihood of pain being attributable to primary dysmenorrhoea.

In some care settings, pelvic examination is advocated in sexually active adolescents with dysmenorrhoea as a routine part of care (47, 54), suggesting rectal examination to look for pelvic pathology if this is not acceptable and/or the young person is not sexually active (122, 261). Rectal examination is proposed as a stratagem to consider the possibility of recto-sigmoid endometriotic nodules or scarring (118). We identified no evidence about the sensitivity of pathology detection or on perceptions of acceptability of trans-rectal examination.

## Research gaps

**Figure 5:**
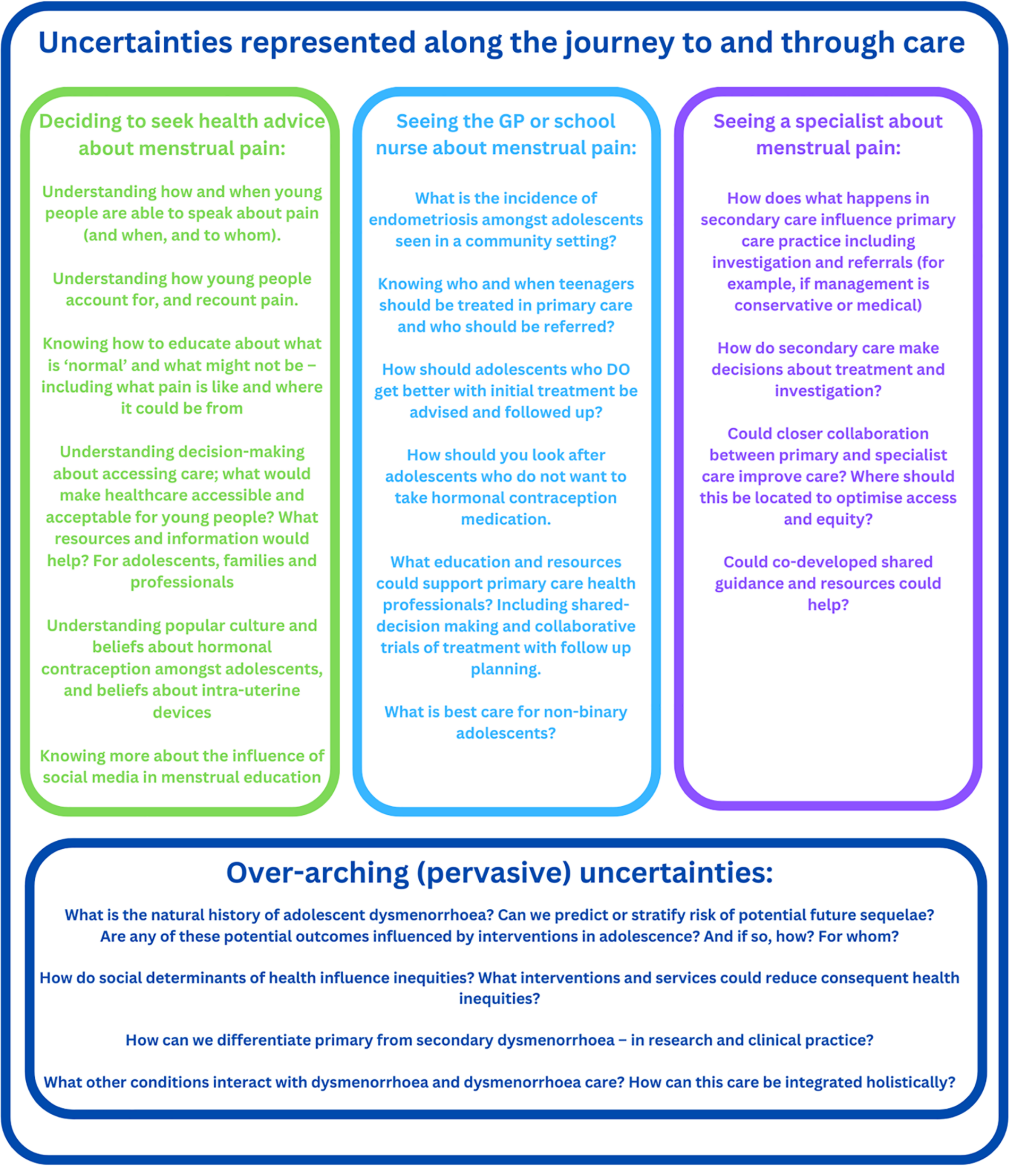
This summarises the research gaps and uncertainties identified in this synthesis and with input from PPI advisers. These are represented along a journey to and through healthcare, situated within the overarching uncertainties identified.

We have summarised possible unanswered questions for exploration and underpinning uncertainties following our review, positioned around a hypothetical journey to and through care in Figure 5. This has been developed with input and guidance from our PPI advisers.

We found a lack of evidence supporting progestogen-only contraception treatments, a gap noted by others (215).

We have also drafted a potential framework for primary care, which supports holding uncertainty and maintaining possibilities about whether pain is primary or secondary during clinical encounters and trials of treatment, and which is embedded in evidence from this systematic review. This is included as Supplementary Appendix E.

## Discussion

### Strengths and limitations

A strength of this synthesis is the breadth of evidence included, and in mapping the type and settings of published evidence. Considering the interface between symptoms and potential diagnoses with pragmatic treatment evidence summaries alongside uncertainties clinicians may encounter situates this review within a general practice setting, where there is little primary empiric evidence.

The focus on adolescence meant we excluded evidence from women in early adulthood. We observed within our searching that there is a significant body of observational evidence undertaken with university students. While there will be many areas of overlap between adolescents (often still in school) and young women at university, it is also possible that there are social and developmental differences between these groups, making the specific focus on this age group appropriate. This evidence synthesis excluded retrospective evidence from adults. The natural history of adolescent dysmenorrhoea is an established research uncertainty. We searched systematically and widely, but may not have identified all potentially relevant papers. Excluding papers written in languages other than English risked missing primary care relevant research or relevant contextual insights and experiences.

### Comparison with other literature

We did not identify research about adolescent dysmenorrhoea conducted within UK general practice, and very few studies within primary healthcare settings, although this is where a majority of UK health contacts occur. There is a known lack of research about adolescent gynaecology, with findings often extrapolated from adult women (306). This is not always appropriate in dysmenorrhoea, given the peak in incidence in adolescence, and within the life course context of the psychosocial development that occurs in adolescence. These concerns were identified by GPs, reflecting on uncertainty about managing adolescent dysmenorrhoea (3).

In a 2022 synthesis taking a bio social approach to representing the life course impacts of dysmenorrhoea, the authors recognise the potential value of a recognised diagnosis in affording validity to pain, to the detriment of those experiencing pain without a “identified pathology” (307). They note that most research considers secondary dysmenorrhoea, and that much research does not effectively delineate between primary and secondary (307). We expand this point by identifying points of tension and inconsistency in the literature which purports to be about primary dysmenorrhoea.

Subsequent systematic reviews have strengthened the evidence for an association between the number and severity of adverse childhood experiences experienced and the likelihood of dysmenorrhoea (308), and between IBS and endometriosis (309) Furthermore, adding to the systematic review reviewing evidence about the association between dysmenorrhoea and chronic pain conditions, there is also evidence about the association between menstrual pain and autonomic dysfunction and bladder pain, potentially starting from menarche (310).

Pain in young people is imperfectly understood and responded to, and there is likely to be a complex and bi-directional relationship between mood and pain (311). However the causal pathways align, if GPs ask about, recognise, and support young people with the pain itself and the emotional impacts of menstrual pain, this is likely to be an important component of care.

The use of Chinese herbal medicine is described as an effective self-care approach (188), and although the conclusions were tempered because of methodological limitations, a Cochrane review, including adolescents, found promising evidence to support the use of Chinese herbal medicine (312).

Evidence in adults, including young adults, supports the potential efficacy of progestogen-only contraception in reducing dysmenorrhoea, including oral desogestrel (313), including in those with dysmenorrhoea associated with endometriosis (314) and the subdermal implant (315).

We did not find qualitative evidence appraising teenagers' views and perspectives on priorities in dysmenorrhoea care. A UK thesis script included interviews with adolescents and mothers and highlighted that both experience uncertainty about when period pain is “normal” and when to ask for advice (316). The interface between representing menstrual pain as normal and seeking care was also highlighted by young adults, including some reporting disquiet about the acceptability of using hormonal contraception to reduce or alter menstrual bleeding (317). Mothers are a frequent source of information and guidance for adolescents with dysmenorrhoea, and so their perspectives and knowledge are important considerations. A 2012 survey study in China of 300 mothers of adolescent daughters identified low levels of knowledge and perceived acceptability of the use of hormonal contraception as a treatment for dysmenorrhoea (318). A survey of parents in Australia also highlighted parental concerns about medication and side effects, including hormonal contraception (319). Given the central place these treatments hold in guidelines for first line empirical therapy, this is important to explore further.

GPs express concern about holding uncertainty when caring for adolescents whose symptoms do improve with a trial of treatment. If symptoms recur if or when they stop hormonal treatment at some later point, and they are subsequently investigated and found to have endometriosis, these care journeys may be characterised as “delayed” diagnoses in retrospective analysis. However, the assumption that they inevitably represent missed opportunities or sub-standard care is overly simplistic (3), including in the context of guidance suggesting referral only if trials of treatment are unsuccessful (109, 270). The complex inter-relationship between exogenous hormones and whether they protect against or exacerbate endometriosis complications and progression remains a critical area of uncertainty (117, 156), which is also a concern for GPs (3).

A family history of dysmenorrhoea predicts dysmenorrhoea (30, 151, 153), and a family history of endometriosis predicts endometriosis (156, 320). NICE Clinical Knowledge Summaries position asking about a family history of dysmenorrhoea as a pointer towards primary dysmenorrhoea (321), and a family history of endometriosis is a risk factor and pointer towards endometriosis. However, noting the well-documented delays and likely incomplete ascertainment of endometriosis diagnoses (322), we consider that asking about a family history of either endometriosis *or* dysmenorrhoea is potentially clinically useful.

With increasing recognition of endometriosis and of the role and value of non-invasive modalities for diagnosis, such as ultrasound or MRI (322), it is hopeful that diagnostic journeys will improve, and require less invasive testing. This would be welcome, not least because diagnostic laparoscopy is associated with both immediate risks but also longer term risks. However, this requires the development of education and resources (including shared-decision making and patient information resources) that recognise the variability of endometriosis, are tailored for adolescents and for community settings, where the prevalence/population is different.

While there is striking uniformity worldwide about the prevalence and impacts of dysmenorrhoea, routes to care and support likely differ, and qualitative perspectives would help shine a light on this, and support the improvements to care and well-being that are demonstrably desperately needed.

This could usefully explore the reasons for school absenteeism, which would enable schools and services to consider if there are adaptations that they could make to facilitate participation in school, including health literacy and self-efficacy, period poverty, menstrual stigma, and access to care.

## Conclusion

Dysmenorrhoea is common and impactful, affecting participation in education and leisure and on well-being. While important to consider whether there is an underlying or associated cause, recognising, validating, and treating pain is essential in it's own right. Earlier and non-invasive diagnostic tools will help us understand more about the community prevalence of endometriosis, and could potentially bring the capacity to make the diagnosis from specialist care into community settings. This is welcome but needs to be accompanied by evidence relevant to the population seen in primary care, with caution about extrapolating risks from tertiary care and specialist clinics (fertility, pelvic pain).

There are evidence-based treatments which can be instigated and supported in general practice, including NSAIDs and treatment with hormonal contraception. However, it is important to ensure care planning and follow up that enables review and re-appraisal of any ongoing concerns or symptoms. Marked symptoms or those not responding to empirical treatment (or if this is not acceptable, tolerated, or is contra-indicated) indicate the need for further assessment and specialist referral. An ultrasound scan can be arranged within general practice, and could identify a structural anomaly or endometrioma, but alone will not exclude endometriosis. There is both a need and an opportunity to develop menstrual education that enables young people to understand how and when to ask for help and advice, and to ensure that there are resources and services for them when they do.

## Data Availability

The original contributions presented in the study are included in the article/**Supplementary Material**, further inquiries can be directed to the corresponding author.
