# Supplementary figures and images for: What is known about adolescent dysmenorrhoea in (and for) community health settings?
Source: Front Reprod Health. 2024 Jul 23;6:1394978. doi: 10.3389/frph.2024.1394978 (PMC11300274; doi:10.3389/frph.2024.1394978)

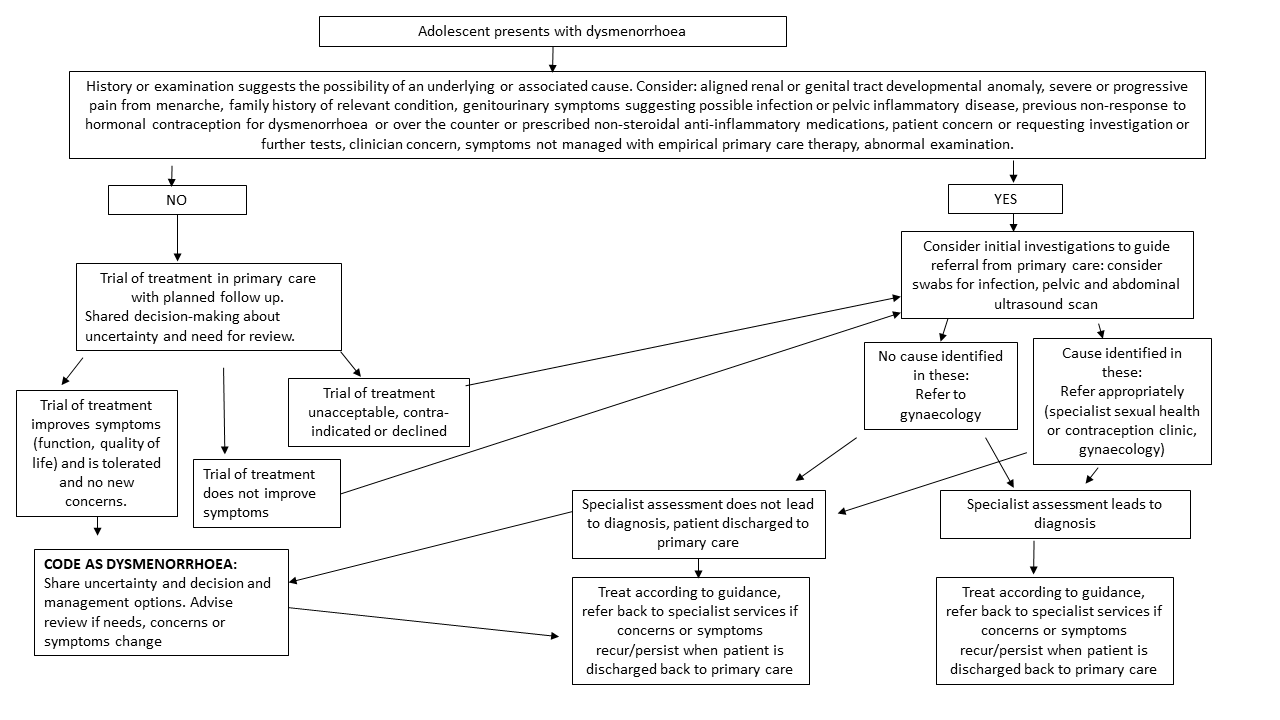

Supplement: Supplementary file 5 [file Image1.tif]
